# Supplementary material for: Development of Dibenzothiazepine Derivatives as Multifunctional Compounds for Neuropathic Pain
Source: Pharmaceuticals (Basel). 2022 Mar 27;15(4):407. doi: 10.3390/ph15040407 (PMC9030932; doi:10.3390/ph15040407)
Supplement: Supplementary file 1 [file pharmaceuticals-15-00407-s001.zip › pharmaceuticals-1629730-supplementary.pdf]

Development of Dibenzothiazepine Derivatives as Multifunctional Compounds for Neuropathic Pain

Young-Hwan Jung [1,†], Yeo Ok Kim [2,†], Koon Mook Kang [1,†], Hyung Gon Lee [2,3], Borum Son [1], Xuehao Han [2,3],
Eunseok Oh [4], Siwon Kim [5], Seon Hee Seo [5], Jong-Hyun Park [5,6], Ki Duk Park [5,6], Woong Mo Kim [2,3], Myung Ha Yoon [2,*] and Yong-Chul Kim [1,7,*]

[1] School of Life Sciences at Gwangju Institute of Science and Technology (GIST), Gwangju 61005, Korea; young-hwan.jung@nih.gov (Y.-H.J.); km1944@gm.gist.ac.kr (K.M.K.); br219@hanmail.net (B.S.)
[2] Department of Anesthesiology and Pain Medicine, Chonnam National University Medical School, Gwangju 61469, Korea; yeok1017@hanmail.net (Y.O.K.); leehg@chonnam.ac.kr (H.G.L.); 752253335h@gmail.com (X.H.); kimwm@chonnam.ac.kr (W.M.K.)
[3] Center for Creative Biomedical Scientists, Chonnam National University Medical School, Gwangju 61469, Korea
[4] Department of Chemistry, Gwangju Institute of Science and Technology (GIST), Gwangju 61005, Korea; dmstjr0130@snu.ac.kr
[5] Convergence Research Center for Brain Science, Korea Institute of Science and Technology (KIST), Seoul 02792, Korea; h15512@kist.re.kr (S.K.); shseo@kist.re.kr (S.H.S.); jhyunprk@kist.re.kr (J.-H.P.); kdpark@kist.re.kr (K.D.P.)
[6] Division of Bio-Medical Science & Technology, KIST School, Korea University of Science and Technology, Seoul 02792, Korea
[7] Department of Biomedical Science and Engineering, Gwangju Institute of Science and Technology (GIST), Gwangju 61005, Korea
* Correspondence: mhyoon@chonnam.ac.kr (M.H.Y.); yongchul@gist.ac.kr (Y.-C.K.)
† These authors contributed equally to this work.

Contents — Pages

Contents	Pages
Synthetic Scheme S1	S2
Chemical synthesis (**18**-**22**)	S3-S4
Experimental details for biology	S5
In vitro metabolic stability of selected compounds	S6
HPLC purity of antiallodynic agents (**12**, **17**)	S7-S8

Scheme S1. Synthesis of compounds **21** and **22**.

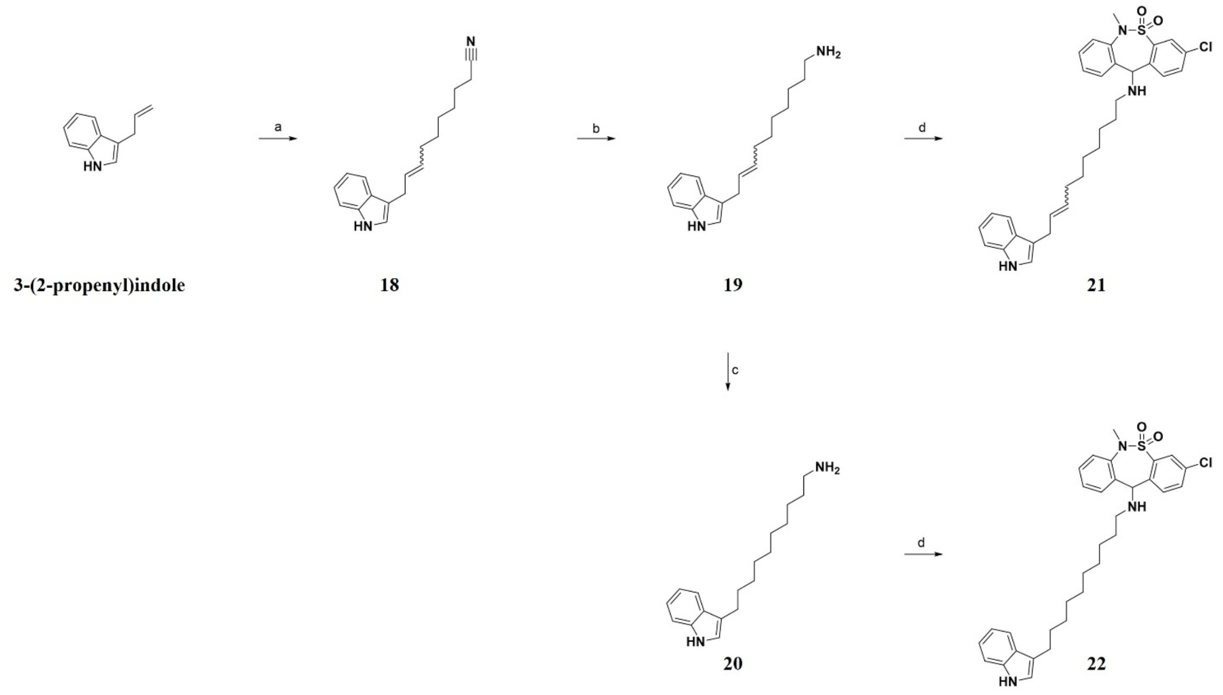

Reagents and conditions. (a) 8-nonenenitrile, Grubbs catalyst (1st generation), DCM, 50 °C, 12 h, 69-75%; (b) $LiAlH_4$, THF, 0 °C, 3.5 h, 48-62%; (c) Pd/C, H_2 gas, MeOH, RT, 0.5 h, 76-85%; (d) 3,11-dichloro-6-methyl-6,11-dihydrodibenzo[c,f][1,2]thiazepine 5,5-dioxide, TEA, DCM, RT, 18 h, 65-78%.

Chemical synthesis

10-(1H-indol-3-yl)dec-8-enenitrile (**18**).

To a mixture of 8-nonenenitrile (1.22 g, 8.91 mmol) and 3-(2-propenyl)indole (0.70 g, 4.45 mmol) in anhydrous dichloromethane, a solution of Grubbs catalyst, 1st generation (0.73 g, 0.89 mmol) in dry dichloromethane was added. After the reaction mixture was stirred for 12 h at 50 °C under an atmosphere of Ar, it was cooled to room temperature. The reaction mixture was diluted with saturated aq. NH_4Cl and extracted with dichloromethane. The combined organic extracts were dried with anhydrous Na_2SO_4, and the mixture was filtered through celite. The filtered mixture was evaporated and purified by silica gel column chromatography in *n*-hexanes/ethyl acetate = 7:1 to afford **18.** Yield 0.85 g (71.8%); ^{1}H NMR ($CDCl_3$, 400 MHz) δ (ppm); 7.57 (d, *J* = 8.0 Hz, 1H), 7.33 (d, *J* = 8.0 Hz, 1H), 7.15-7.20 (m, 1H), 7.05-7.13 (m, 1H), 6.92-7.00 (m, 1H), 5.45-5.75 (m, 2H), 3.50 (m, 2H), 2.25-2.35 (m, 2H), 1.95-2.05 (m, 2H), 1.55-1.70 (m, 2H), 1.25-1.36 (m, 6H); MS (ESI): [M+H] = 267.1.

10-(1H-indol-3-yl)dec-8-en-1-amine (**19**).

To a solution of **18** (0.20 g, 0.75 mmol) in tetrahydrofuran at 0 °C, a suspension of 1M $LiAlH_4$ in tetrahydrofuran (2.25 mL, 2.25 mmol) was added dropwise over a period of 0.5 h. After the addition was complete, the reaction mixture was stirred at 0 °C under N_2 atmosphere for 3 h. The reaction mixture was quenched with saturated aqueous NH_4Cl, and then the mixture was filtered through celite. The filtered mixture was evaporated and purified by silica gel column chromatography in chloroform/methanol = 10:1 to afford **19.** Yield 0.16 g (52.2%); ^{1}H NMR (CD_3OD, 400 MHz) δ (ppm); 7.43-7.48 (m, 1H), 7.25-7.33 (m, 1H), 7.00-7.07 (m, 1H), 6.90-6.97 (m, 2H), 5.45-5.70 (m, 2H), 3.35-3.50 (m, 2H), 2.81-2.87 (m, 2H), 1.97-2.05 (m, 2H), 1.55-1.65 (m, 2H), 1.26-1.42 (m, 8H); MS (ESI): [M+H] = 271.1.

10-(1H-indol-3-yl)decan-1-amine (**20**).

To a solution of **19** (25.0 mg, 0.092 mmol) in methanol was added 10% Pd/C (5.0 mg). The reaction mixture was stirred for 0.5 h under H_2 atmosphere. After completion of reaction, the mixture was filtered through celite pad. The filtrate was evaporated and purified by silica gel column chromatography in chloroform/methanol = 20:1 to afford **20.** Yield 20 mg (79.4%); ^{1}H NMR (CD_3OD, 400 MHz) δ (ppm); 7.44-7.48 (m, 1H), 7.26-7.29 (m, 1H), 7.00-7.07 (m, 1H), 6.90-

6.97 (m, 2H), 2.81-2.87 (m, 2H), 2.67-2.73 (m, 2H), 1.55-1.73 (m, 4H), 1.24-1.42 (m, 12H); MS (ESI): [M+H] = 273.1.

11-((10-(1H-indol-3-yl)dec-8-en-1-yl)amino)-3-chloro-6-methyl-6,11-dihydrodibenzo[c,f][1,2]thiazepine 5,5-dioxide (**21**).

Following the general procedure A, **21** was synthesized from **19** (20.0 mg, 0.073 mmol). Yield 32.1 mg (77.2%); ^{1}H NMR ($CDCl_3$, 400 MHz) δ (ppm); 7.93-7.96 (m, 1H), 7.41-7.51 (m, 3H), 7.29-7.40 (m, 4H), 7.14-7.22 (m, 2H), 7.04-7.09 (m, 1H), 6.97-7.02 (m, 1H), 5.35-5.55 (m, 2H), 4.96 (s, 1H), 3.33 (s, 3H), 2.91-2.94 (m, 2H), 2.40-2.45 (m, 2H), 1.82-1.97 (m, 2H), 1.35-1.50 (m, 2H), 1.15-1.30 (m, 8H); MS (ESI): [M+H] = 562.9; HRMS (ESI) $[M+H]^+$ ($C_{32}H_{36}ClN_3O_2S$): calcd. 562.2290, found. 562.2251.

11-((10-(1H-indol-3-yl)decyl)amino)-3-chloro-6-methyl-6,11-dihydrodibenzo[c,f][1,2]thiazepine 5,5-dioxide (**22**).

Following the general procedure A, **22** was synthesized from **20** (6.0 mg, 0.022 mmol). Yield 8.2 mg (66.1%); ^{1}H NMR ($CDCl_3$, 400 MHz) δ (ppm); 7.94 (d, *J* = 2.0 Hz, 1H), 7.40-7.50 (m, 3H), 7.31-7.40 (m, 4H), 7.26-7.30 (m, 1H), 7.16-7.19 (m, 1H), 6.97-7.10 (m, 2H), 4.98 (s, 1H), 3.35 (s, 3H), 2.62-2.72 (m, 2H), 2.42-2.48 (m, 2H), 1.43-1.53 (m, 4H), 1.17-1.32 (m, 12H); MS (ESI): [M+H] = 564.9; HRMS (ESI) $[M+H]^+$ ($C_{32}H_{38}ClN_3O_2S$): calcd. 564.2394, found. 564.2398.

Experimental details for Biology

Cell culture. HEK293 cells stably expressing human DA transporter (HEK-hDAT), human NE transporter (HEK-hNET) and human serotonin transporter (HEK-hSERT) were kindly provided by Professor Bryan Roth (University of North Carolina at Chapel Hill). And HEK293 cells constitutively expressing human κ- or μ- opioid receptor (HEK-hKOR_NM000912 or HEK-hMOR_NM000914) were constructed by Professor Ki Duk Park (in Korea Institute of Science and Technology). And CHO-K1 OPRM1 β-Arrestin cell line (#93-0213) was purchased from Europins DiscoveRx (Fremont, CA, USA). Cells were maintained in Dulbecco's modified Eagle's medium (HyClone, Logan, UT, USA) supplemented with 10%(v/v) fetal bovine serum (FBS, HyClone, Logan, UT, USA), penicillin (100 U/mL), and streptomycin (100 μg/mL) in the presence of Geneticin G418 (350 μg/mL, 200 μg/mL, 500 μg/mL, 1000 μg/mL and 1000 μg/mL for HEK-hDAT, HEK-hNET, HEK-hSERT, HEK-hKOR and HEK-hMOR respectively) in a humidified 5% CO2 incubator at 37 ˚C. In case of both HEK-hKOR and HEK-hMOR, cells were additionally maintained in 0.2 mg/mL hygromycin B, 1 μg/mL puromycin B, 1× non-essential amino acids. The cells were subcultured every 3-4 days.

Microsomal stability. The metabolic stability of compounds was evaluated in rat liver microsomes (RLM). Microsomes (0.5 mg/ml) and test compound (1 μM) in 100 mM potassium phosphate buffer (pH 7.4) were preincubated with shaking (100 rpm) for 5 min at 37 °C. The metabolic reaction was initiated by the addition of an NADPH generating system (60 mM potassium phosphate buffer pH 7.4, 1.3 mM NADP, 3.3 mM glucose-6-phosphate, 0.3 μL glucose-6-phosphate dehydrogenase (0.4 U/mL), and 3.3 mM magnesium chloride) and incubated at 37 °C. Aliquots of 100 μL were withdrawn at 0, 10, 20, 30 and 60 min, and 2 volumes of ice-cold acetonitrile (ACN) were added to terminate the reaction. After mixing by vortex and centrifugation (14000 rpm) for 15 min at 4 °C, the supernatant was transferred to liquid chromatography vials and analyzed by an LC-MS/MS system.

Table S1. In vitro metabolic stability of selected compounds.

**Compounds**	**Recovery (%)[a]**		
	**Human**	**Rat**	**Mouse**
**12**	**5%**	**6%**	**5%**
**17**	**0%**	**1%**	**1%**

[a]% remaining after 30 min incubation with S9 microsomal fraction.

Figure S1. HPLC purity of **12** (98%, R_t = 19.56 min) as an antiallodynic agent.

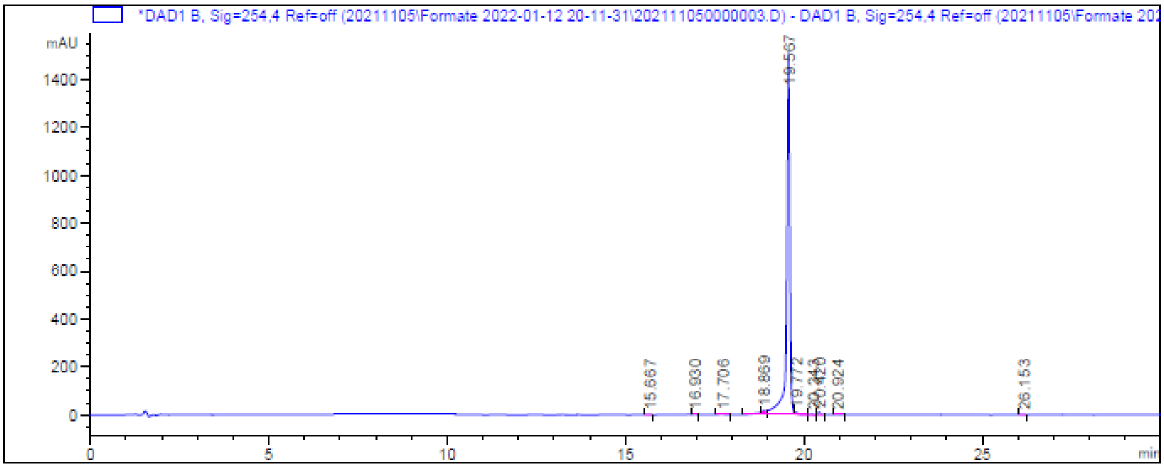

==

Area Percent Report

==

Sorted By : Signal
Multiplier : 1.0000
Dilution : 1.0000
Do not use Multiplier & Dilution Factor with ISTDs

Signal 1: DAD1 B, Sig=254,4 Ref=off
Signal has been modified after loading from rawdata file!

Peak #	RetTime [min]	Type	Width [min]	Area [mAU*s]	Height [mAU]	Area %
1	15.667	BB	0.1024	10.10456	1.54305	0.0978
2	16.930	BB	0.0660	8.36063	1.95054	0.0809
3	17.706	BB	0.1852	15.36503	1.17018	0.1488
4	18.869	VV E	0.0725	24.37649	5.22166	0.2360
5	19.567	VV R	0.1003	1.01530e4	1513.31213	98.2919
6	19.772	VB E	0.0830	38.66914	6.73508	0.3744
7	20.243	BB	0.0874	13.02500	2.32399	0.1261
8	20.420	BB	0.0801	55.06189	10.69470	0.5331
9	20.924	BB	0.0802	5.35812	1.03926	0.0519
10	26.153	BB	0.0931	6.11439	1.00323	0.0592

Figure S2. HPLC purity of **17** (95%, R_t = 14.52 min) as an antiallodynic agent.

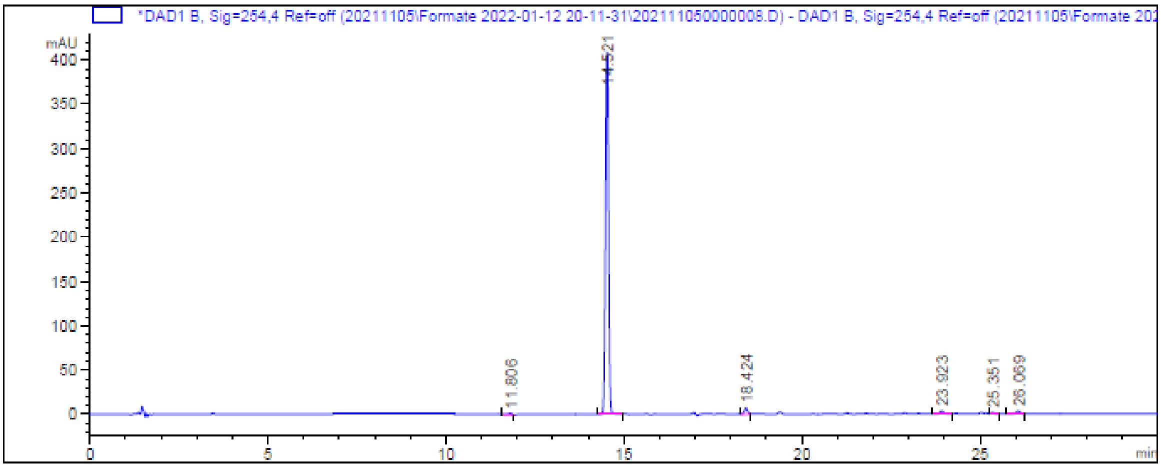


```
=====================================================================
                        Area Percent Report
=====================================================================

Sorted By             :      Signal
Multiplier            :      1.0000
Dilution              :      1.0000
Do not use Multiplier & Dilution Factor with ISTDs


Signal 1: DAD1 B, Sig=254,4 Ref=off
 Signal has been modified after loading from rawdata file!

Peak RetTime Type  Width     Area      Height     Area
  #   [min]        [min]    [mAU*s]     [mAU]       %
----|-------|----|-------|----------|----------|--------|
   1  11.806 BB    0.1292   13.59158    1.56694   0.5495
   2  14.521 BB    0.0890 2351.59839  409.33325  95.0737
   3  18.424 BB    0.0928   40.11567    6.79968   1.6219
   4  23.923 BB    0.1168   29.75666    3.74103   1.2030
   5  25.351 VB    0.1061   12.27415    1.78730   0.4962
   6  26.069 BB    0.1141   26.11046    3.53890   1.0556
```
